# Genome-Wide Identification and Characterization of DNA Methylation and Long Non-Coding RNA Expression in Gastric Cancer

**DOI:** 10.3389/fgene.2020.00091

**Published:** 2020-02-27

**Authors:** Peng Song, Lei Wu, Wenxian Guan

**Affiliations:** ^1^ Department of General Surgery, Nanjing Drum Tower Hospital, The Affiliated Hospital of Nanjing University Medical School, Nanjing, China; ^2^ Department of Laboratory Medicine, The First Affiliated Hospital of Nanjing Medical University, Nanjing, China

**Keywords:** DNA methylation, long non-coding RNA, epigenetics, prognosis, gastric cancer

## Abstract

Abnormal DNA methylation, an epigenetic modification, has increasingly been linked to the pathogenesis of many human cancers. However, there has been little focus on the DNA methylation patterns of genes encoding long noncoding RNAs (lncRNAs) in gastric cancer (GC). This study comprehensively determined DNA methylation and lncRNA expression profiles in GC through genome-wide analysis. Differentially methylated loci and lncRNAs were identified by integrating multi-omics data. In total, 548 differentially methylated CpG sites in lncRNA promoters and 2,399 differentially expressed lncRNAs were screened that were capable of distinguishing GC from normal tissues. Among them, 22 differentially methylation sites in 17 lncRNAs were inversely related to expression levels. Further analysis of DNA methylation status and gene expression level in GC revealed that three CpG sites (cg01550148, cg22497867, and cg20001829) and two lncRNAs (RP11-366F6.2 and RP5-881L22.5) were significantly associated with GC patient overall survival. Molecular function analysis showed that these abnormally methylated lncRNAs were mainly involved in transcriptional activator activity. Our study identified several lncRNAs regulated by aberrant DNA methylation that have clinical utility as novel prognostic biomarkers in GC. These findings help improve the understanding of methylated patterns of lncRNAs and further our knowledge of the role of epigenetics in cancer development.

## Introduction

Gastric carcinoma (GC) is the fourth most prevalent malignancy and third leading cause of cancer death worldwide (Torre et al., 2015). Histologically, GC demonstrates marked heterogeneity at the cytologic level, resulting in the classification of tumor subtypes. Distinct molecular genetic profiles, morphology, and expression of specific markers have been used to investigate the diversity and characteristics of GC (Zouridis et al., 2012; Cancer Genome Atlas Research Network, 2014). Therefore, identifying potential biomarkers to further understand the pathogenesis of GC is critical.

Long noncoding RNAs (lncRNAs) are loosely defined as RNAs more than 200 bases in length with no apparent coding capacity (Mattick and Rinn, 2015). LncRNAs regulate gene expression at transcriptional and post-transcriptional levels and thus are involved in diverse biological functions. Furthermore, recent studies have demonstrated a role for lncRNAs in carcinogenesis (Spizzo et al., 2012; Zhuo et al., 2019). DNA methylation, a key epigenetic mechanism, plays a crucial role in the regulation of gene expression, genomic imprinting, genome stabilization, and chromatin modification. Aberrant DNA methylation has been reported to be involved in the formation and progressions of diseases, especially cancers (Guo et al., 2018; Xu et al., 2019). Recent studies have showed that expression alterations of lncRNA-encoding genes mediated by changes in methylation can subsequently affect their downstream targets. For instance, the lncRNA C5orf66-AS1 functions as a tumor suppressor gene in GC, and aberrant hypermethylation of the regions around its transcription start site (TSS) is associated with its expression and is cancer-specific (Guo et al., 2018). This study indicated that hypermethylation of the C5orf66-AS1 promoter may serve as a potential prognostic marker in predicting GC patient survival. Shahabi et al. identified an epigenetically deregulated lncRNA linc00261, whose expression was lost in lung adenocarcinoma through DNA methylation silencing. The authors found that linc00261 acted upstream of ATM activation to facilitate DNA damage response activation and its loss resulted in malignant phenotypes and predisposed lung cells to cancer development (Shahabi et al., 2019). Additionally, lncRNAs showing aberrant DNA methylation may serve as potential epigenetically-based diagnostic factors. Silencing from CpG-island methylation of promoter-induced transcribed ultraconserved regions (T-UCRs) is common in many tumors and linked to colorectal cancer diagnosis (Kottorou et al., 2016; Honma et al., 2017). Therefore, elucidating the relationship between DNA methylation and lncRNA expression is essential for understanding GC development and potentially identifying new prognostic or diagnostic markers.

Here, we employed multigenomic data from The Cancer Genome Atlas (TCGA) and Gene Expression Omnibus (GEO) datasets to systematically characterize global DNA methylation levels, lncRNA expression profiles, and clinical features in GC. Our results decode the landscape of DNA methylation-mediated regulation for lncRNAs and provide promising biomarkers in the diagnosis and treatment of GC.

## Methods

### DNA Methylation and Gene Expression Data

The DNA methylation array data (Illumina Infinium Human Methylation27, 450 BeadChip) were downloaded from the UCSC Xena browser (https://xenabrowser.net/). A Human Methylation27 BeadChip array of GC (GSE30601) was obtained from the GEO database (https://www.ncbi.nlm.nih.gov/geo/). Level-3 RNA-sequencing data (HTSeq-Counts and HTSeq-FPKM-UQ) and the clinicopathological and survival data of GC patients were also downloaded from the Xena website.

### Analysis of DNA Methylation Data

Differentially methylated CpG sites (DMCs) and differentially methylated regions (DMRs) between GC samples and adjacent tissues were identified using the minfi package (Version: 1.32.0; http://www.bioconductor.org/packages/release/bioc/html/minfi.html) (Fortin et al., 2017). Bump hunting method was applied to identify DMRs. False discovery rate (FDR) was calculated from multiple testing corrections of raw P-value by the Benjamini and Hochberg method (Benjamini et al., 2001). The genomic annotation of each CpG site was conducted using the hm27.hg38.manifest file (http://zwdzwd.io/InfiniumAnnotation). The coordinates of the individual lncRNA were extracted from GENCODE v22 (https://www.gencodegenes.org/human/release_22.html). After the preprocessing the coordinates of CpG sites and lncRNAs, we further integrated both information based on the genomic location, considering differentially methylated loci within promoter regions (DNA sequences between –2,500 and 1,000 bp relative to the putative TSS). Manhattan plot was constructed to depict the distribution of CpG sites according to FDR *via* qqman package (Version: 0.1.4; https://cran.r-project.org/web/packages/qqman/index.html) (Turner, 2018).

### Differential Long Non-Coding RNA Expression Analysis

Read count tables were imported into the edgeR package for identifying differentially expressed transcripts (Version: 3.7, https://bioconductor.org/packages/release/bioc/html/edgeR.html) (McCarthy et al., 2012). LncRNA catalogue was retrieved from GENCODE v22. Genes with FDR < 0.05 and absolute fold change (FC) > 2 were considered differentially expressed lncRNAs (DElncs).

### Integrated Analysis of DNA Methylation and Long Non-Coding RNA Expression

The correlation analysis between DMCs and DElncs was calculated and those with |coefficient of correlation| > 0.3 and P-value < 0.05 were considered significant. The visualization of the potential regulation of CpG sites to genes was constructed in Cytoscape 3.7.1 (Shannon et al., 2003).

### Functional Annotation and Enrichment Analysis for Long Non-Coding RNAs

ClusterProfiler tool (Version: 3.8.1, https://bioconductor.org/packages/release/bioc/html/clusterProfiler.html) was used to perform Gene Ontology (GO) function and Gene Set Enrichment Analysis (GSEA) for DElncs with the DMCs (Yu et al., 2012). Spearman's correlation coefficients of expression levels between DElncs and protein-coding RNAs were calculated. The deregulated protein-coding genes were considered for GO analysis, setting parameters as “pAdjustMethod” = “BH,” “pvalueCutoff” = 0.05, and “qvalueCutoff” = 0.05 for multiple comparisons. Terms from GO (molecular function) database slice were tested for enrichment. Mappings between GO terms and Entrez Gene IDs relied on the regularly updated R package org.Hs.eg.db (Version 3.10.0; https://bioconductor.org/packages/release/data/annotation/html/org. Hs.eg.db.html). The value of log_2_ (FC) calculated by edgeR package was used as ranking metric for GSEA. We used the canonical pathways sub-collection of the C2 collection in the Molecular Signatures Database as the gene sets in the analysis. The leading-edge subset of genes in an enriched gene set are defined as those that appear in the ranked list before the point at which the running sum reaches its maximum deviation from zero.

### Statistical Analysis

Cox proportional hazard regression analyses were carried out to compare clinical features, DNA methylation, DElncs expression, and GC patients' prognosis. Survival curves were compared using Kaplan-Meier Plotter with log-rank test. All statistical analysis was two-sided and P < 0.05 was defined as statistically significant. Statistical analysis was performed using R programming language v.3.5.3.

## Results

### Characteristics of the DNA Methylation Pattern in Gastric Cancer

Because the TCGA-450k set contained only two normal samples and the number was too small to reach statistical significance with respect to determining the DNA methylation profile of GC, we used the TCGA-27k set to identify DMCs and DMRs. We obtained 6,404 CpG sites with FDR < 0.05 between 48 GC and 25 non-tumor samples and identified 1,078 DMCs with a delta-beta value > 0.2. A total of 103 DMRs were identified based on the following parameters: resamples = 100, cut off = 0.2, and probe number ≥ 2. The 103 DMRs included 65 hypermethylated regions and 38 hypomethylated regions.

**Figure 1:**
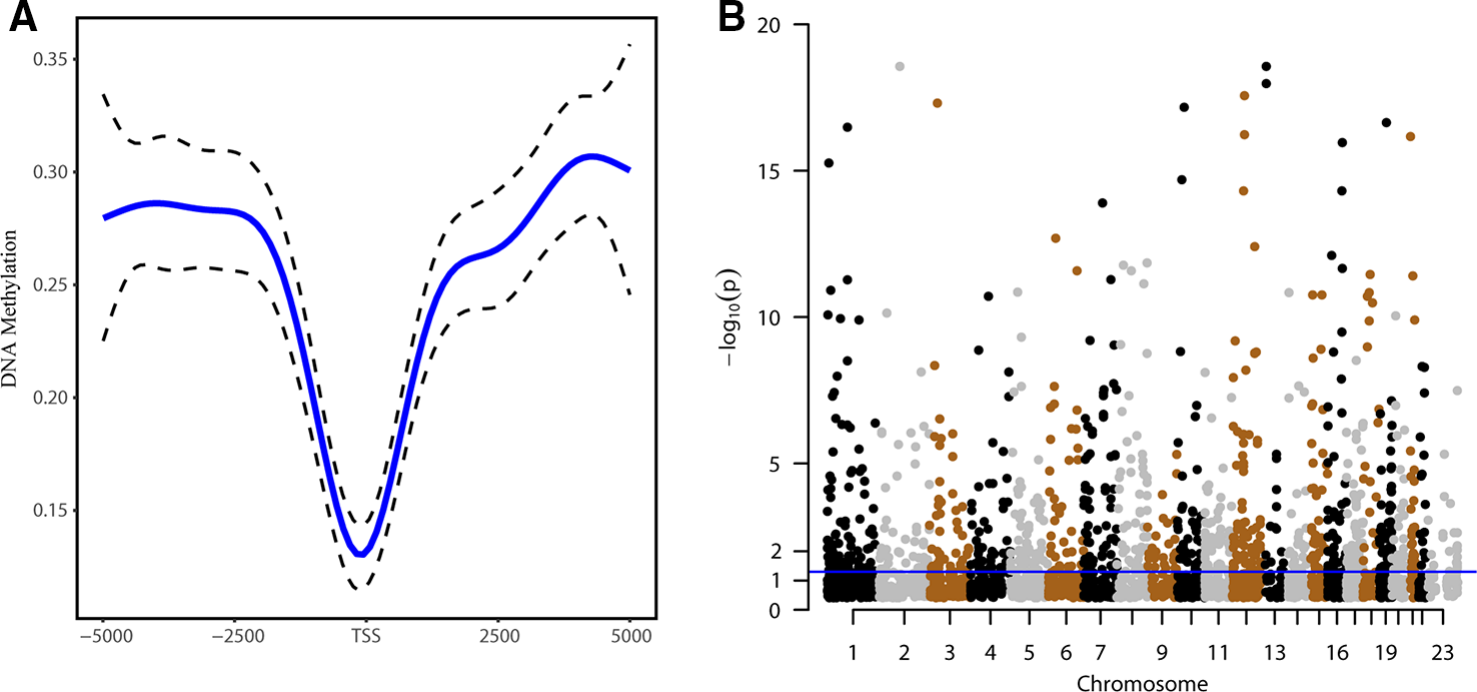
DNA methylation patterns of genes encoding long noncoding RNAs (lncRNAs). **(A)** Distribution of the methylation levels around lncRNA genes in sperm ranging from 5 kb upstream to 5 kb downstream of the transcription start site (TSS). **(B)** Manhattan plot of CpG sites in the promoter regions of lncRNA genes; dots above the blue line indicate CpG sites with P value < 0.05.

To identify DNA methylation alterations in lncRNA promoter regions, 3,010 CpG sites were examined. The methylation distribution in lncRNAs showed a V-shaped curve around the TSSs, indicating a relative reduction of the methylation density at the TSS (**Figure 1A**). As shown by Manhattan plot (**Figure 1B**), the CpG sites were distributed in all chromosomes, and 698 probes were found using the threshold of FDR < 0.05. We subsequently validated the DNA methylation patterns of the CpG sites of interest in an independent cohort (GSE30601) and found that 548 probes overlapped with the 698 sites reported in TCGA-27k set (**Supplementary Table S1**).

### Characteristics of Long Non-Coding RNA Expression in Gastric Cancer

**Figure 2:**
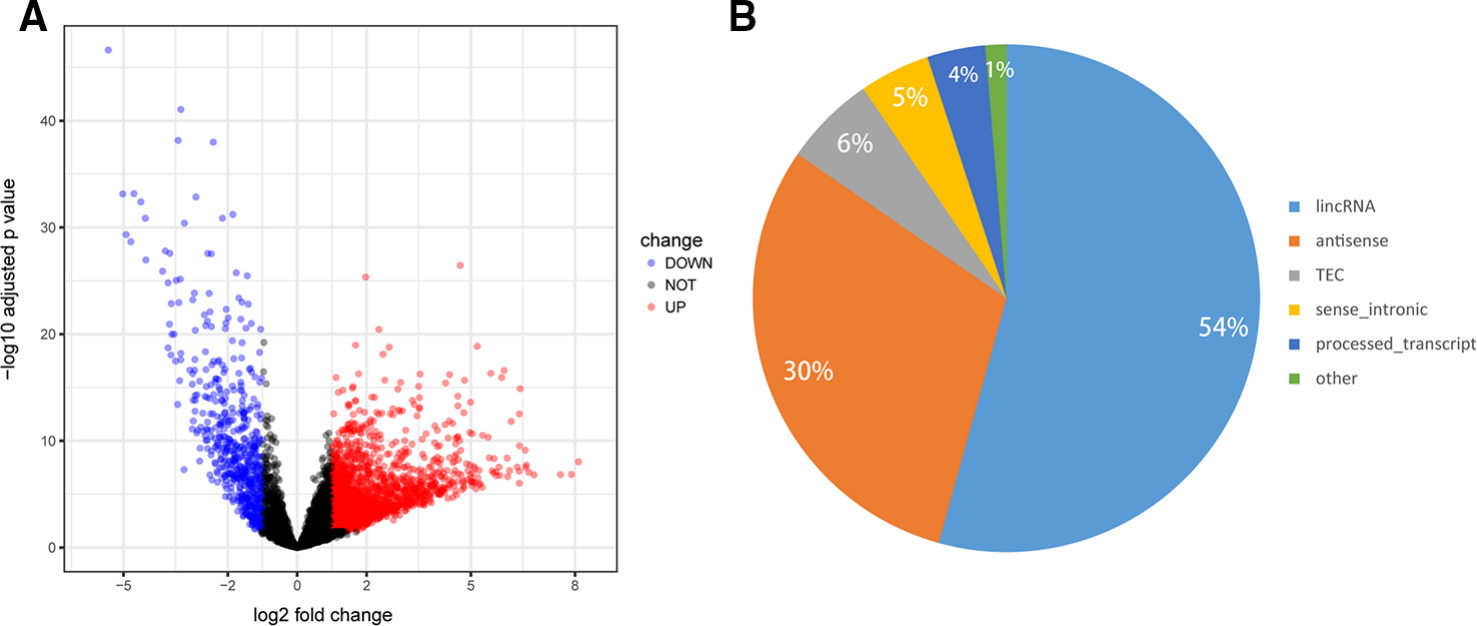
Differential expression profiles of long noncoding RNAs (lncRNAs) in gastric cancer (GC). **(A)** Volcano plot of the differentially expressed lncRNAs between GC tumors and normal tissues. The red points represent lncRNAs that are significantly upregulated in GC while blue points represent downregulated lncRNAs (absolute FC >2 and FDR < 0.05). **(B)** Pie chart shows the number of differentially expressed lncRNAs in each category.

To determine the lncRNA expression profile in GC, RNA-seq data of 375 GC tumors and 32 normal tissues were retrieved from TCGA. Among the 6,820 lncRNAs, we identified 2,399 DElncs, including upregulated 1,830 lncRNAs and 569 downregulated lncRNAs, using the criteria of FDR < 0.05 and absolute FC > 2 (**Figure 2A**, **Supplementary Table S2**). We then analyzed the categories of the 2,399 DElncs, as shown in **Figure 2B**. Long intergenic non-coding RNAs (lincRNAs) accounted for 54.3% of all DElncs, followed by antisense transcripts (30.3%). The remaining non-coding transcript types were sense_intronic transcripts (4.5%), processed_transcripts (3.7%), and sense_overlapping transcripts (1.3%).

### Integrated Analysis of Differential Methylation and Long Non-Coding RNA Expression Data

**Figure 3:**
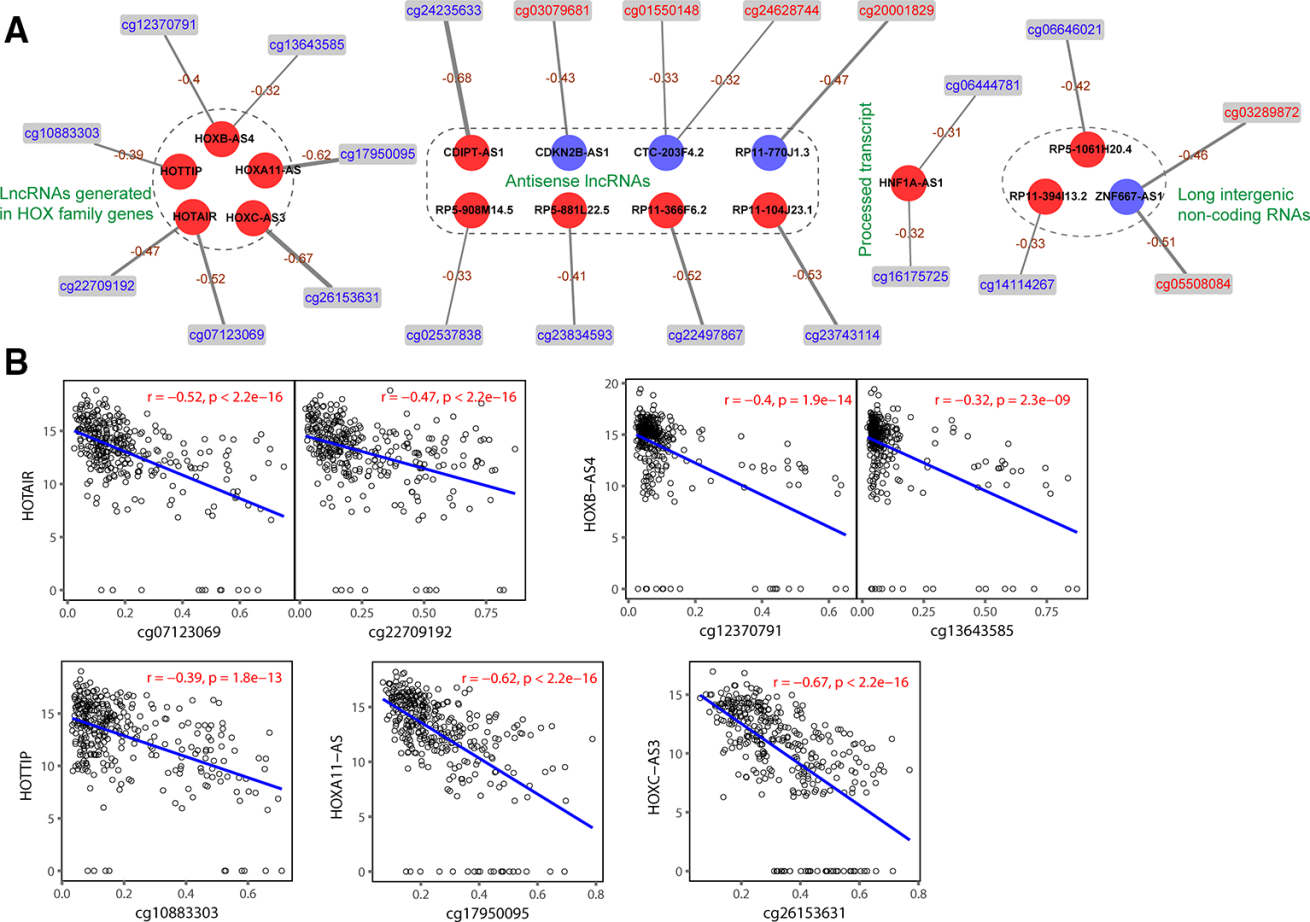
Relation between DNA methylation and long noncoding RNA (lncRNA) expression. **(A)** Correlation between differentially methylated CpGs (DMCs) and lncRNAs. Circles and rectangles represent lncRNAs and DMCs, respectively. Red color indicates upregulated or hypermethylated, and blue indicates downregulated or hypomethylated. **(B)** Correlation (P values derive from Spearman's correlation) between DNA methylation and the expression of HOX family genes associated with five lncRNAs in matched samples.

After the profiles of DNA methylation and lncRNA expression were preprocessed, we combined the two omics data for further analysis. By associating the 548 DMCs to 2,399 DElncs, 31 negative correlated pairs and 1 positive correlated pair were obtained in TCGA-27k set. DNA methylation in promoters is well known to negatively correlate with corresponding gene expression (Mosquera Orgueira, 2015). The 31 negative correlated pairs were validated in TCGA-450k set, and 22 probes showed a significantly inverse correlation with the promoter methylation of 17 aberrantly expressed lncRNAs (**Supplementary Table S3**). We used the negatively correlated pairs to construct a DNA methylation-regulated network that was composed of 39 nodes, including 6 hypermethylated DMCs, 16 hypomethylated DMCs, 13 upregulated lncRNAs, and 4 downregulated lncRNAs (**Figure 3A**). As shown in **Figure 3B**, the expression of 5 lncRNAs (HOTAIR, HOTTIP, HOXA11-AS, HOXB-AS4, and HOXC-AS3) generated in HOX family genes were negatively correlated with their methylation levels. The functions of these 17 lncRNAs are listed in **Supplementary Table S4**; only 6 of the lncRNAs have been reported to function GC (Zhang et al., 2014; Liu et al., 2015; Sun et al., 2016; Wu et al., 2017; Liu et al., 2018; Zhang et al., 2018; Song et al., 2019).

### Impact of DNA Methylation and Long Non-Coding RNA Expression on Gastric Cancer Survival

**Figure 4:**
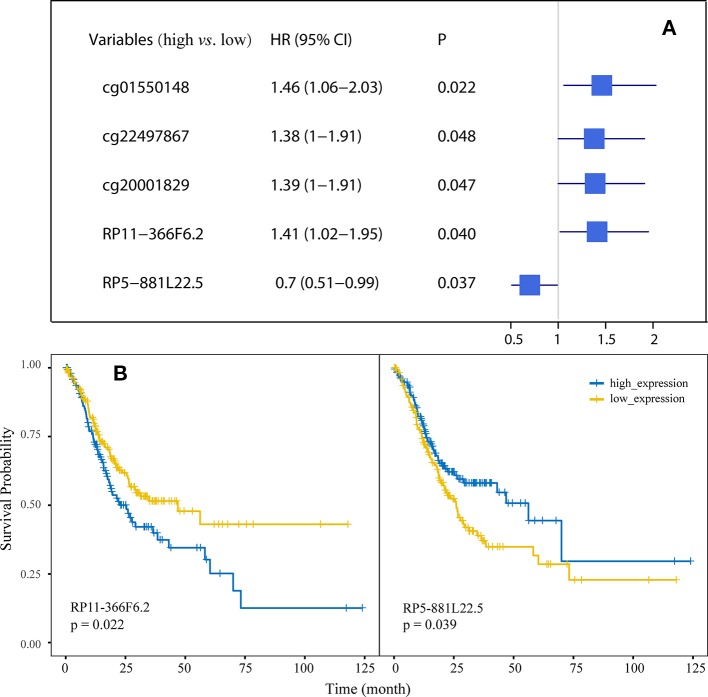
Association of the methylation of differentially methylated CpGs (DMCs) and expression of differentially expressed long noncoding RNAs (DElncs) with survival of GC patients. **(A)** Forest plot depicting correlations between the methylation of DMCs with the survival of GC patients, using the median expression of probes as the cut-off value. **(B)** Kaplan-Meier analysis of overall survival for GC patients according to RP11-366F6.2 and RP5-881L22.5 expression.

Univariate Cox regression was used to evaluate the association of the 22 probes and 17 lncRNAs with overall survival in GC and the results identified three CpG sites (cg01550148, cg22497867, and cg20001829) and two lncRNAs (RP11-366F6.2 and RP5-881L22.5) with P < 0.05. Forest plot demonstrated that the methylation of the three probes and two lncRNAs were associated with the overall survival time of GC patients (**Figure 4A**). **Figure 4B** shows the Kaplan-Meier curves for survival in GC patients according to RP11-366F6.2 and RP5-881L22.5 expression. High expression of RP11-366F6.2 was significantly correlated with poor survival compared with low expression. Additionally, poor survival was observed for patients with low expression of RP5-881L22.5 compared with patients with high levels (P = 0.039). In the multivariate Cox analysis, even after adjustment by tumor stage and other covariates, the expression of the two lncRNAs was still significantly associated with patient survival (P < 0.05, **Table 1**).

**Table 1:** Univariate and multivariate Cox regression analysis of variables associated with gastric cancer (GC) patient survival.

Variables	Univariate analysis			Multivariate analysis		
N=351	HR	95% CI	P	HR	95% CI	P
Age (≥67/ < 67)	1.44	1.04–2.00	0.029	1.49	1.06–2.11	0.023
Sex (male/female)	1.33	0.93–1.89	0.115	1.31	0.91–1.89	0.151
Tumor_stage (III+IV/I+II)	1.85	1.29–2.63	<0.001	1.89	1.32–2.70	<0.001
RP11-366F6.2 (high/low) *	1.41	1.02–1.95	0.040	1.45	1.03–2.05	0.033
RP5-881L22.5 (high/low) ^†^	0.70	0.51–0.99	0.037	0.68	0.49–0.96	0.030

HR, hazard ratio; CI, confidence interval.

*Using the mean expression of genes as the cut-off value.

^†^ Using the median expression of genes as the cut-off value.

### Association of Deregulated Long Non-Coding RNAs With Biological Pathways and Processes

**Figure 5:**
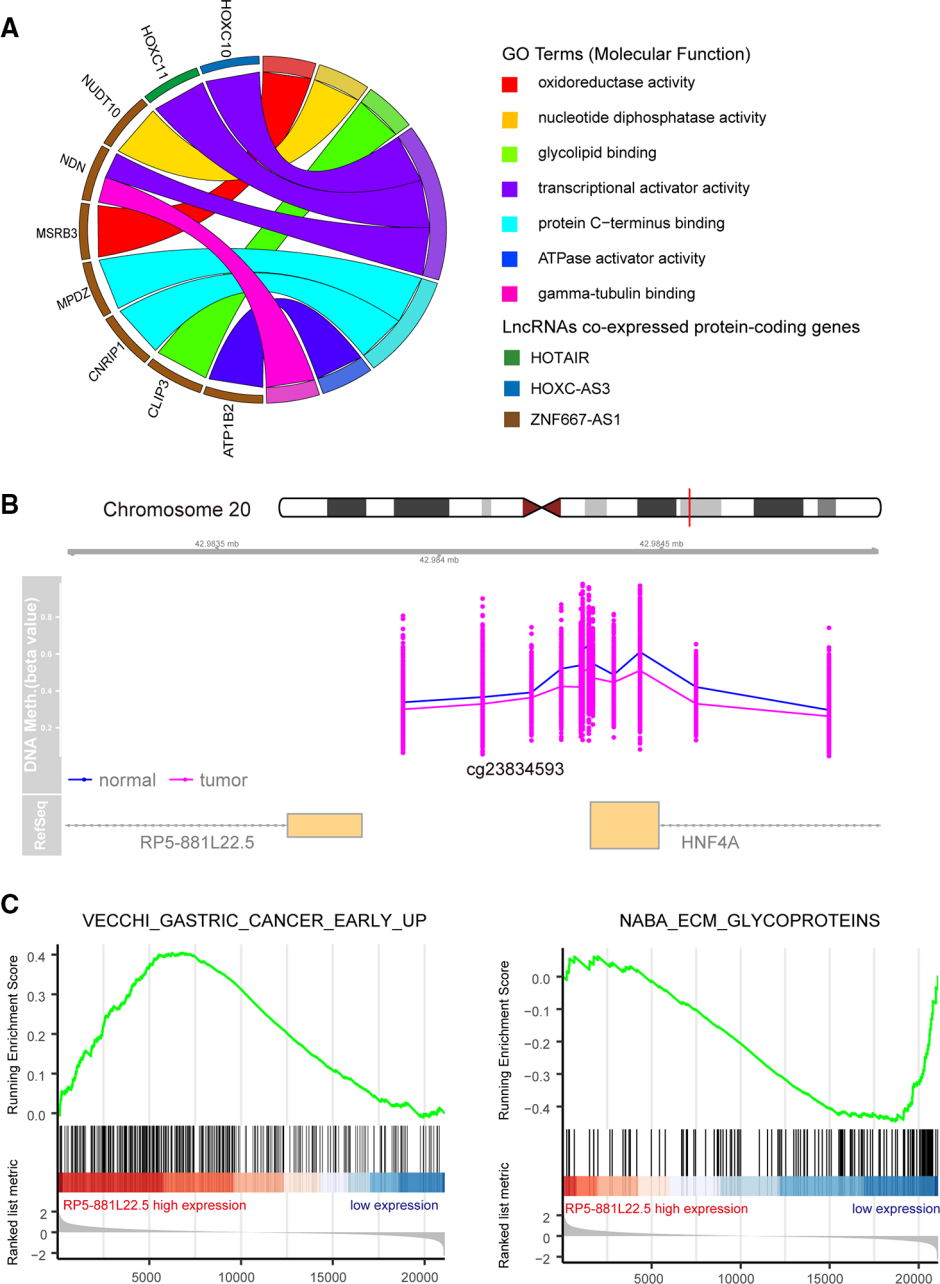
Functional annotation for differentially methylated differentially expressed long noncoding RNAs (DElncs). **(A)** Circular plot of molecular function regulator Gene Ontology Term. **(B)** RP5-881L22.5 promoter hypermethylation in gastric cancer (GC) tumors compared with normal tissues. **(C)** Gene set enrichment analysis of RP5-881L22.5 in the The Cancer Genome Atlas (TCGA) dataset.

To better understand the biological function of the 17 DElncs, we constructed a co-expression network of deregulated protein-coding genes and lncRNAs. Using the Spearman's correlation coefficient above 0.8, a total of 32 deregulated mRNAs co-expressed with five lncRNAs (ZNF667-AS1, RP5-881L22.5, HOTAIR, HOTTIP, and HOXC-AS3) were acquired for GO enrichment analysis. Only nine protein-coding genes associated with HOTAIR, HOXC-AC3, and ZNF667-AS1 were assigned GO molecular function, involving oxidoreductase activity, nucleotide diphosphatase activity, and transcriptional activator activity (**Figure 5A**). In addition, GSEA analysis was performed to identify the associated biological processes and signaling pathways for these deregulated lncRNAs. As an example, we explored a functionally unknown lncRNA, RP5-881L22.5, with significantly hypomethylated promoter regions that was upregulated in GC tissue and implicated with prognosis (**Figure 5B**). The expression of RP5-881L22.5 was positively correlated with “VECCHI_GASTRIC_CANCER_EARLY_UP” set, in which upregulated genes could differ early GC and normal tissue samples. The “NABA_ECM_GLYCOPROTEINS” set was enriched in the RP5-881L22.5 low expression group, which implied that this lncRNA could suppress tumor metastasis (**Figure 5C**).

## Discussion

Cancer involves a complex regulatory network, and therefore integrating multiple omics data is required in the era of precision medicine (Olivier et al., 2019). The increasing applications of multi-omic profiling of GC have delivered new insight into the dynamics of this cancer type. In this study, we characterized DNA methylation in the promoters of lncRNA-encoding genes and inferred the potential lncRNAs regulated by aberrant DNA methylation in GC. Differential analyses were performed to compare DNA methylation and gene expression patterns between GC and normal tissues, and 548 DMCs and 2,399 DElncs were obtained. We thus identified lncRNAs (such as HOTAIR, HOTTIP, HOXA11-AS, HOXB-AS4, and HOXC-AS3) that could be regulated by aberrant DNA methylation *via* combination analysis. We further divided the potentially epigenetic regulated lncRNAs into different groups to explore their biological and clinical relationships with GC and found that the expressions of RP11-366F6.2 and RP5-881L22.5 were related to the prognosis of GC.

Methylation that interferes with transcription machinery binding to DNA has been reported to be highly associated with repression of gene transcription (Suzuki and Bird, 2008). Our research showed that a large number of lncRNAs were epigenetically deregulated by promoter methylation, and lncRNAs were globally hypomethylated, which was consistent with the previous observation that increased global DNA hypomethylation was a major event for the development and progression of cancer (Zeng et al., 2017). HOX genes are a subset of evolutionarily conserved homeobox genes that encode a class of important transcription factors that function in numerous developmental processes (Quinonez and Innis, 2014). By characterizing the transcriptional landscape of the four human HOX loci (A–D) at five base pair resolution in 11 anatomic sites, Rinn et al. identified 231 HOX non-coding RNAs (Rinn et al., 2007). HOTAIR is located in the HOXC cluster and serves as a scaffold protein by binding Polycomb repressive complex 2 (PRC2, including SUZ12, EED, and EZH2) *via* its 5′-domain and the LSD1/CoREST/REST complex *via* its 3′-domain, mediating gene silencing and reprogramming the overall chromatin dynamics in GC (Qu et al., 2019). Like HOTAIR, HOXA11-AS recruits EZH2 along with the histone demethylase LSD1 or DNMT1, which promotes proliferation and invasion of GC (Sun et al., 2016). HOTTIP enhances the expression of neighboring HOXA genes, particularly HOXA13 (Chang et al., 2016). HOXC-AS3, an antisense transcript of HOXC10, mediates gene transcriptional regulation in the tumorigenesis of GC by binding to YBX1 (Zhang et al., 2018). Genome-wide screening isolated HOXB-AS4 as specifically methylated in pancreatic cancer cells, which was useful to assess a cancer cell fraction in DNA samples (Ishihara et al., 2018).

Therapeutic targets and prognosis prediction from a comprehensive analysis of multi-omics data and clinical profiles is a critical for better understanding the biological complexity of GC. We identified two hypomethylated DElncs (RP11-366F6.2 and RP5-881L22.5) in GC that were significantly associated with overall survival. RP11-366F6.2, also called MAGEA4-AS1, is located in chrX and was reported to have significantly high expression in several tumor tissues, such as breast cancer and laryngeal squamous cell carcinoma (Yuan et al., 2017; Liu and Ye, 2019). Although pre-ranked GSEA analysis for RP11-366F6.2 returned no significantly gene sets, examining the functional roles of deregulated genes (such as MAGEA4, MAGEA10, HOXD10 and IGF2BP1) in the leading edge set indicated RP11-366F6.2 might be associated with tumor invasion and metastasis (Suzuki et al., 2008; Schultz-Thater et al., 2011; Xu et al., 2019). Regarding RP5-881L22.5, Zhu et al. developed an eleven-lncRNA signature, including this lncRNA, which could provide an effective individual mortality risk prediction and risk stratification in GC patients (Zhu et al., 2018). However, the biological functions of RP5-881L22.5 have not been determined. GSEA results revealed that RP5-881L22.5 was likely to be involved in an extracellular matrix (ECM) interaction pathway. Glycoproteins make the ECM a cohesive network of molecules by linking cells together with structural components (Nallanthighal et al., 2019). Adhesive glycoproteins can bind to ECM components to activate downstream signaling pathways to regulate epithelial-mesenchymal transition, self-renewal, migration, differentiation, and proliferation (Naba et al., 2016; Song et al., 2017). For example, the adhesion of cancer cells to fibronectin, a major adhesive ECM glycoprotein, remodels the tumor vasculature, enhances tumorigenicity, and facilitates metastasis. This mechanism could partly explain why decreased RP5-881L22.5 expression indicated a poor prognosis for GC patients.

To investigate the effect of epigenetically deregulated lncRNA in biological processes and pathways, an integrated analysis of DElncs and predicted mRNAs expression was performed. GO analysis revealed that these lncRNAs were involved in dysregulated transcriptional programs that invariably lead to cancer. We also found that predicted mRNAs HOXC10 and MSRB3 in GO analysis were significantly associated with overall survival in GC patients (Kim et al., 2019; Ma et al., 2019).

Several limitations in the present study should be pointed out. First, integrated analysis of genome-wide DNA methylation and lncRNA expression was based on the 27K Illumina array platform, which only contained 27,578 individual registered probes, and thus some possibly important methylation differences may be lacking from the current results. Second, the results of the present study are preliminary and primarily derived from bioinformatics analysis, and lack functional validation of the epigenetically deregulated lncRNAs. Third, due to limited availability of clinical data, it was not possible to obtain deeper insights into characterizing phenotype-genotype relationships.

In conclusion, the present results provide evidence for the changes of widespread DNA methylation of lncRNA-encoding genes in GC patients. The candidate factors identified in this study might function as potential molecular phenotypic biomarkers, especially RP11-366F6.2 and RP5-881L22.5, which were associated with prognosis. Our results help elucidate a more detailed explanation of epigenetic mechanisms for GC and deepen our understanding of the aberrantly methylated patterns in lncRNA-encoding genes.

## Data Availability Statement

The following information was supplied regarding data availability:.

The level 3 TCGA data for DNA methylation arrays and lncRNA expression are available in Xena website.

HumanMethylation27: https://gdc.xenahubs.net/download/TCGA-STAD.methylation27.tsv.gz
HumanMethylation450: https://gdc.xenahubs.net/download/TCGA-STAD.methylation450.tsv.gz
HTSeq-Counts: https://gdc.xenahubs.net/download/TCGA-STAD.htseq_counts.tsv.gz
HTSeq-FPKM-UQ: https://gdc.xenahubs.net/download/TCGA-STAD.htseq_fpkm-uq.tsv.gz
Phenotype: https://gdc.xenahubs.net/download/TCGA-STAD.GDC_phenotype.tsv.gz
Survival data: https://gdc.xenahubs.net/download/TCGA-STAD.survival.tsv.gz

The microarray data that support this study are available through the NCBI database under accession GSE30601.

The data used to support the findings of this study are included within the article.

## Author Contributions

PS and WG designed the study. PS and LW collected, analysed and interpreted the data. PS and LW wrote the draft. PS and WG edited the manuscript.

## Conflict of Interest

The authors declare that the research was conducted in the absence of any commercial or financial relationships that could be construed as a potential conflict of interest.
