# Supplementary material for: Genome-Wide Identification and Characterization of DNA Methylation and Long Non-Coding RNA Expression in Gastric Cancer
Source: Front Genet. 2020 Feb 27;11:91. doi: 10.3389/fgene.2020.00091 (PMC7056837; doi:10.3389/fgene.2020.00091)
Supplement: Supplementary file 3 [file Table_3.doc]

**Supplementary Table S3**: Association between DNA methylation and long noncoding RNAs expression

| Probe | LncRNA | TCGA-27k set | |  | TCGA-450k set | |
| --- | --- | --- | --- | --- | --- | --- |
|  |  | r | P value |  | r | P value |
| cg24235633 | CDIPT-AS1 | -0.680 | 3.64E-06 |  | -0.684 | 5.29E-48 |
| cg03079681 | CDKN2B-AS1 | -0.331 | 0.045244 |  | -0.427 | 2.25E-16 |
| cg24628744 | CTC-203F4.2 | -0.472 | 0.003217 |  | -0.321 | 1.61E-09 |
| cg01550148 | CTC-203F4.2 | -0.459 | 0.004316 |  | -0.330 | 5.13E-10 |
| cg06444781 | HNF1A-AS1 | -0.652 | 1.21E-05 |  | -0.306 | 9.62E-09 |
| cg16175725 | HNF1A-AS1 | -0.642 | 1.81E-05 |  | -0.323 | 1.21E-09 |
| cg07123069 | HOTAIR | -0.438 | 0.006698 |  | -0.517 | 1.70E-24 |
| cg22709192 | HOTAIR | -0.434 | 0.007281 |  | -0.472 | 3.91E-20 |
| cg10883303 | HOTTIP | -0.505 | 0.001426 |  | -0.386 | 1.78E-13 |
| cg17950095 | HOXA11-AS | -0.740 | 1.69E-07 |  | -0.619 | 4.22E-37 |
| cg13643585 | HOXB-AS4 | -0.438 | 0.006737 |  | -0.318 | 2.33E-09 |
| cg12370791 | HOXB-AS4 | -0.325 | 0.049774 |  | -0.400 | 1.88E-14 |
| cg26153631 | HOXC-AS3 | -0.636 | 2.31E-05 |  | -0.673 | 6.88E-46 |
| cg23743114 | RP11-104J23.1 | -0.343 | 0.037933 |  | -0.527 | 1.36E-25 |
| cg22497867 | RP11-366F6.2 | -0.538 | 0.000597 |  | -0.518 | 1.32E-24 |
| cg14114267 | RP11-394I13.2 | -0.522 | 0.00092 |  | -0.329 | 5.55E-10 |
| cg20001829 | RP11-770J1.3 | -0.548 | 0.000442 |  | -0.473 | 3.16E-20 |
| cg06646021 | RP5-1061H20.4 | -0.547 | 0.000457 |  | -0.417 | 1.27E-15 |
| cg23834593 | RP5-881L22.5 | -0.528 | 0.000789 |  | -0.409 | 4.62E-15 |
| cg02537838 | RP5-908M14.5 | -0.652 | 1.26E-05 |  | -0.333 | 3.24E-10 |
| cg05508084 | ZNF667-AS1 | -0.719 | 5.28E-07 |  | -0.514 | 3.37E-24 |
| cg03289872 | ZNF667-AS1 | -0.464 | 0.003843 |  | -0.461 | 3.61E-19 |

r: correlation coefficient
