# Supplementary material for: Genome-Wide Identification and Characterization of DNA Methylation and Long Non-Coding RNA Expression in Gastric Cancer
Source: Front Genet. 2020 Feb 27;11:91. doi: 10.3389/fgene.2020.00091 (PMC7056837; doi:10.3389/fgene.2020.00091)
Supplement: Supplementary file 4 [file Table_4.doc]

**Supplementary Table S4**: Expression and role of some long noncoding RNAs in gastric cancer

| LncRNA | Role | Reference |
| --- | --- | --- |
| CDKN2B-AS1 | - upregulated in GC tissues - can recruit and bind to PRC2, epigenetic repression of miR-99a/miR-449a in Trans to control the targets--mTOR and CDK6/E2F1 pathway | Zhang et al., 2014 |
| HNF1A-AS1 | - upregulated in GC tissues - EGR1 can directly bind to the promoter of HNF1A-AS1 - function as competing endogenous RNA by binding to miR-661, upregulating the expression of CDC34 - regulate CDK2, CDK4, cyclin E1 and p21 to affect GC cell proliferation and cell-cycle progression | Liu et al., 2018 |
| HOTAIR | - upregulated in GC tissues - can recruit and bind to PRC2, epigenetically represses miR34a to controls the targets C-Met (HGF/C-Met/Snail pathway) and Snail - promote epithelial-mesenchymal transition (EMT) by switching histone H3 lysine 27 acetylation to methylation at the E‑cadherin promoter | Liu et al., 2015  Song et al., 2019 |
| HOTTIP | - contribute to HOXA13-mediated BMP7 expression in GC cancer cells and induce pluripotent stem cells (iPSCs) | Wu et al., 2017 |
| HOXA11-AS | - upregulated in GC tissues - function as a scaffold, recruit EZH2 along with the histone demethylase LSD1 or DNMT1 - function as a molecular sponge for miR-1297 | Sun et al., 2016 |
| HOXC-AS3 | - upregulated in GC tissues - activate by gain of H3K4me3 and H3K27ac - interact with YBX1 | Zhang et al., 2018 |
| CDIPT-AS1  CTC-203F4.2  HOXB-AS4  RP11-104J23.1  RP11-366F6.2  RP11-394I13.2  RP11-770J1.3  RP5-1061H20.4  RP5-881L22.5  RP5-908M14.5  ZNF667-AS1 | - not report in GC |  |

GC: gastric cancer
